# Cost-Effectiveness Assessment of Internet of Things in Smart Cities

**DOI:** 10.3389/fdgth.2021.662874

**Published:** 2021-05-24

**Authors:** Nuria Febrer, Frans Folkvord, Francisco Lupiañez-Villanueva

**Affiliations:** ^1^Open Evidence Research Group, Universitat Oberta de Catalunya, Barcelona, Spain; ^2^Tilburg School of Humanities and Digital Sciences, Communication and Cognition, Tilburg University, Tilburg, Netherlands; ^3^Faculty of Information and Communication Science, Universitat Oberta de Catalunya, Barcelona, Spain

**Keywords:** IoT, Smart Cities, Cost-effectiveness, modeling, evaluation

## Abstract

With the ongoing rapid urbanization of city regions and the growing need for (cost-)effective healthcare provision, governments need to address urban challenges with smart city interventions. In this context, impact assessment plays a key role in the decision-making process of assessing cost-effectiveness of Internet of Things–based health service applications in cities, as it identifies the interventions that can obtain the best results for citizens' health and well-being. We present a new methodology to evaluate smart city projects and interventions through the MAFEIP tool, a recent online tool for cost-effectiveness analysis that has been used extensively to test information and communications technology solutions for healthy aging. Resting on the principles of Markov models, the purpose of the MAFEIP tool is to estimate the outcomes of a large variety of social and technological innovations, by providing an early assessment of the likelihood of achieving anticipated impacts through interventions of choice. Thus, the analytical model suggested in this article provides smart city projects with an evidence-based assessment to improve their efficiency and effectivity, by comparing the costs and the efforts invested, with the corresponding results.

## Introduction

The ongoing rapid urbanization of city regions urges governments to face and come up with innovative ideas to overcome urban challenges and support and improve the health of citizens and healthcare for patients, whereby digitalization is considered to be a useful method to reinforce efficiency and reduce costs (1). For example, the World Health Organization (WHO) Healthy Cities Network and associated national networks have established hundreds of member cities globally that aim to benefit from Internet of Things (IoT) to improve the health and well-being of citizens (2, 3). Although during the last few years smart cities have been established across Europe and other continents, limited research has been conducted to evaluate smart city interventions and their impact and other outcomes of embedded smart technologies for cities and citizens and their health and well-being (4). A smart city is a place where digital and telecommunication technologies (e.g., IoT services and applications for healthcare, active and healthy aging technologies, algorithms, and artificial intelligence methods for gather relevant knowledge from IoT devices) are used to benefit inhabitants and businesses (2). In a recent systematic review performed by (5), they showed that the most relevant applications supported by a smart city infrastructure with a significant impact on healthcare provision were applications for population surveillance, active aging, healthy lifestyles, disabled people, response to emergencies, better service organization, and socialization.

In this context, we present a case study that focuses on better service organization to reduce pollution to decrease the adverse effects that mobility behavior has on citizens health, which describes the potential benefits of using the MAFEIP (Monitoring and Assessment Framework for the European Innovation Partnership) tool to evaluate smart city projects and interventions by using a cost-effectiveness analysis that has been used extensively in the health economy sector. The MAFEIP tool was originally developed to assess the impact and cost-effectiveness of digital health interventions and is highly promising to be used for different digital interventions as well, such as smart city projects.

The purpose of the MAFEIP tool is to estimate the outcomes of a large variety of social and technological innovations, by providing an early assessment of the likelihood that interventions will achieve the anticipated impact. In addition, the MAFEIP tool also helps to identify what drives interventions' effectiveness or efficiency in order to guide further design, development, or evaluation. MAFEIP therefore represents a clear support to the decision-making process, also for smart city projects and interventions.

The MAFEIP tool rests on the Markov model principle, which is an analytical decision-making model developed by and for health economics (6–9). The main objective of the MAFEIP tool is to provide support in the decision-making process, including an *ex ante* analysis before a concrete intervention is implemented. The Markov model is able to tackle uncertainty in the real effects and costs, and its flexibility allows for the analysis of a large and heterogeneous range of interventions. The model uses the best evidence available from multiple sources, such as administration records, official databases, *ad hoc* information collected for projects' evaluation, or results from evaluations in similar interventions.

Furthermore, the MAFEIP tool provides a useful methodology for the early assessment of the likelihood that interventions will achieve the anticipated impact, while at the same time helping to identify what drives interventions' effectiveness or efficiency in order to guide further design, development, or evaluation. Thus, it can be helpful for a wide variety of stakeholders involved in the IoT-based health service applications in cities. First, health or social care providers can use the MAFEIP tool for codesigning technology-based solutions, while also using the evidence from real life pilots to assess the effectiveness, impact, and utility, to make a more informed decision to invest or to buy a specific technology-based solution. Second, the MAFEIP tool has been shown to be a valuable instrument in Health Technology and Intervention Assessment to inform policy decision making in relation to citizens' health and well-being (10, 11). The tool is able to analyze with more precision the value of the innovation for citizens and other stakeholders and support the systematic evaluation of properties, effects, and/or impacts of health technologies and interventions in different population target groups. Third, big companies, small and midsize enterprises, and startups can take advantage of MAFEIP's utility in assessing the potential impact of new business propositions for healthcare interventions and thus guiding the decision-making process for further technology developments. Last, the MAFEIP tool can be relevant to researchers, as it can be used to improve the quality and relevance of future research and to better serve the information needs of citizens, payers, and other decision makers by helping to identify gaps in evidence.

## Materials and Equipment

The MAFEIP monitoring framework is an online and free-to-use tool that rests on the principles of decision analytic modeling. The MAFEIP tool is based on a traditional Markov model, which is an approach that is common in health economic evaluations and mostly used to assess the impact of healthcare innovations in terms of health outcomes and resource use. Based on the data introduced in the tool, which may be (preliminary) data from clinical studies, expert opinions and user's own views, or data based on a randomized controlled trial, this model performs an incremental analysis of the impact of innovations. As a result, users need data on both the current care situation for the target population and the situation in which the intervention is used (12).

In order to run the model, users need to introduce to MAFEIP different parameters divided in four sections, namely, (1) model analysis, (2) costs associated with health states and intervention costs, (3) transition probabilities for moving between states with and without the intervention, and (4) utilities (also called quality-of-life weights) that are associated with each state. A value has to be selected for each input parameter in order to run the model. Subsequently, the model runs the analyses by itself and provides graphical and numerical outcomes of the analyses.

## Methods

### Markov Model

**Figure 1:**
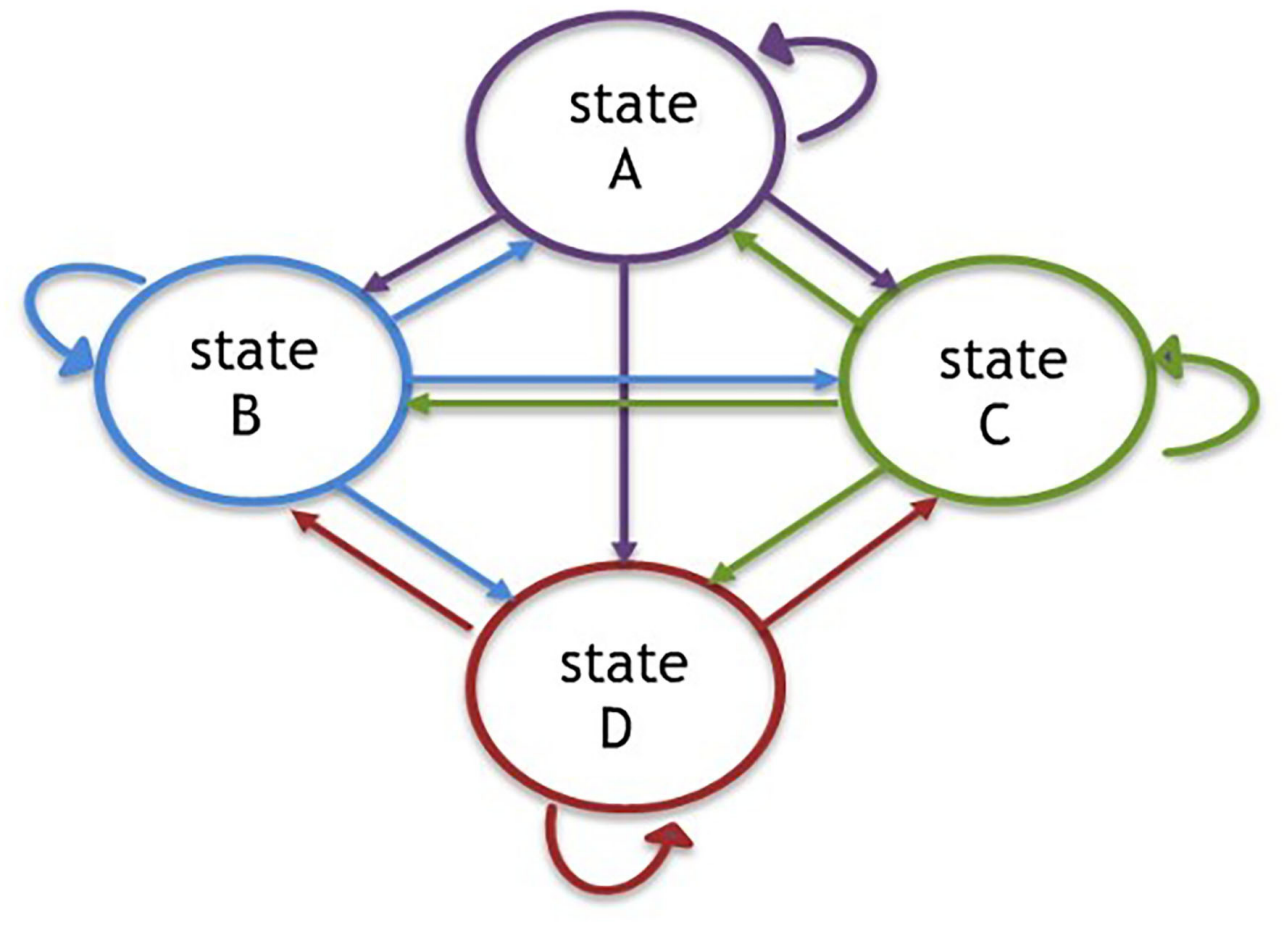
Four states of Markov model.

The MAFEIP tool rests on the principles of Markov models, based on the definition of a specific number of states (see a four-state example on Figure 1), to which certain costs and effects are defined. These effects can be measured with different indicators, depending on the intervention and the objective pursued. One of the key points of this particular model is that it measures the “transition,” meaning that it calculates the probability of “population” (which could be defined as citizens, houses, neighborhoods, sensors, buses, etc.) moving from one state to another one. The model can also take into account the duration of the cycles, by introducing the frequency of these transitions (e.g., monthly, annual, etc.), as well as the total number of cycles of the simulation. For instance, if one wants to conduct an evaluation in 5, 10, or 20 years, the MAFEIP tool provides tailored opportunities to evaluate one's smart city intervention project.

### Parameters and Incremental Cost-Effectiveness

Costs, effects, and probabilities of transition constitute the main parameters of the model, and they must be specified both with and without the implementation of the evaluated intervention (actual situation in case of an evaluation *ex ante*, counterfactual, etc.). Based on these, the simulation compares both situations^1^ and presents as a main result the incremental cost-effectiveness (ICE). It is calculated for a specific period of time, keeping in mind that the probability of being in each state and all the respective costs and effects. For example, if in period 0 we are in “A” scenario, and we assumed that all population is in the same situation, the associated cost for this period would be C_A_, and the effect, E_A_. If the odds of reaching states B, C, and D in period 1 are, respectively, 0.4, 0.2, and 0.1 (and 0.3 of remain on state A), the cost value of period 1 would be:


$$\begin{array}{l} {\text{C}}_{1}=\left(0.3{\text{C}}_{\text{A}}+0.4{\text{C}}_{\text{B}}+0.2{\text{C}}_{\text{C}}+\;0.1{\text{C}}_{\text{D}}\right) \end{array}$$

And the effect value is:


$$\begin{array}{l} {\text{E}}_{1}=\left(0.3{\text{E}}_{\text{A}}+0.4{\text{E}}_{\text{B}}+0.2{\text{E}}_{\text{C}}+\;0.1{\text{E}}_{\text{D}}\right) \end{array}$$

**Figure 2:**
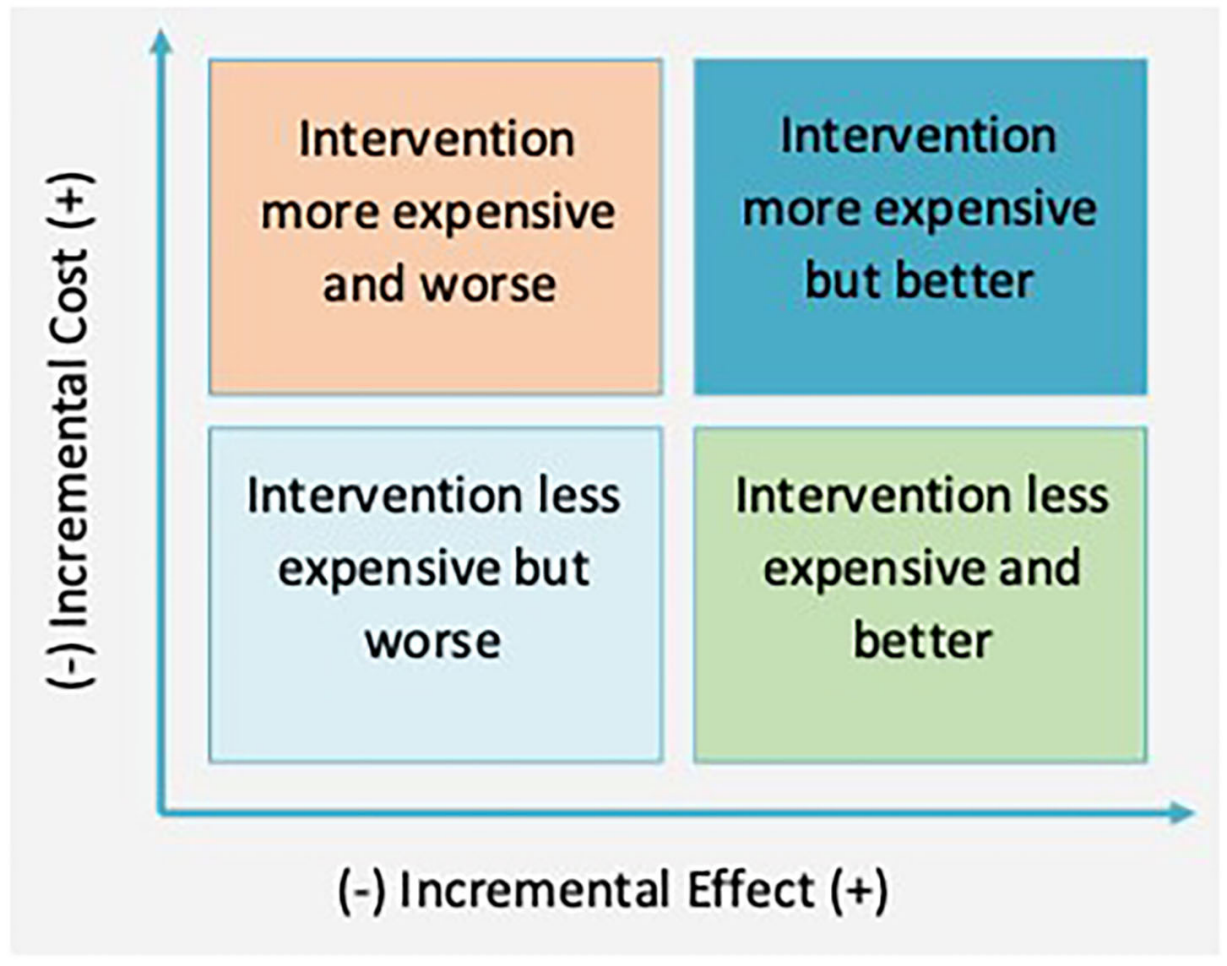
Cost-effectiveness table.

**Figure 3:**
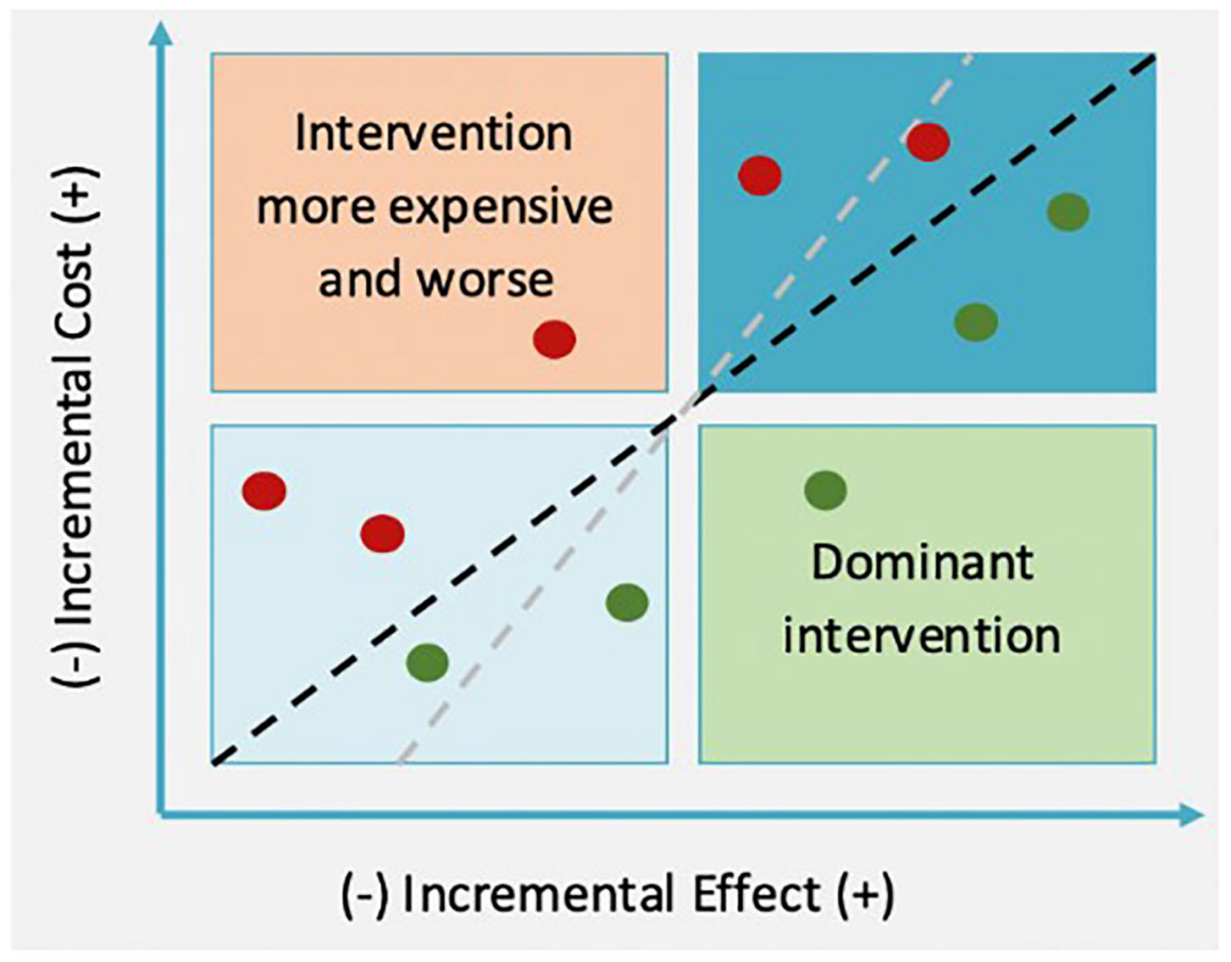
Cost-effectiveness table and ICE.

For each period, these values are calculated and included in the evaluation, and they are compared between *non-intervention* and *intervention* situations. Subtracting *non-intervention* costs and effects from *intervention* values, we obtain the ICE. The ICE is the ratio of these two and indicates the cost of getting one effect unit; for example, the avoidable death cost or reduction of a CO_2_ ton emission. ICE provides information regarding the suitability of implementing a concrete intervention. The visualization of the ICE can be seen in Figures 2, 3.

The ICE might be in four quadrants, depending on cost and effect differences between *intervention* and *non-intervention*. On the top left quadrant, intervention is dominant; it is more expensive and less effective than the alternative one, and therefore, it should not be implemented. On the other hand, if the ICE falls within the bottom left quadrant, the intervention dominates, and it must be applied as it is cheaper and more effective than the initial situation. In terms of the other two quadrants, the decision is less clear. Within the top right quadrant, the intervention is not only more effective but also more expensive. Regarding the bottom left quadrant, the intervention is cheaper, but less effective. In these two cases, the decision is determined by willingness to pay (WTP); therefore, a project should be implemented if the ICE is lower than the WTP (discontinue lines).

An intervention should be accepted when ICE < WTP, which are shown as the green points in Figure 3. However, an intervention would not be accepted by the ICEs defined by the red points. If WTP was bigger, the line would be steeper (gray line). In that case, if an *intervention* was more effective than a *non-intervention* (top right quadrant), it would be more likely for the ICE point to be placed below the WTP line.

### Case Study Using the MAFEIP Tool

The next paragraphs describe the procedure to apply the model through a hypothetical intervention: install sensors in the city to improve public services delivered by buses to reduce the pollution and thereby reduce adverse health outcomes (e.g., respiratory infections, heart disease, lung cancer) (13). The first step of the evaluation is to define the intervention and its objectives. In this case, the intervention purpose would be defined as follows:

“*Improve public transport organization with sensors that measure traffic, the number of users on each zone, etc. to increment its use and with the final purpose of decreasing pollution levels and reduce adverse health outcomes.”*

Related to this, it is useful to conduct a context analysis. This analysis describes the different actors involved in the intervention, the main beneficiaries, the elements that can influence its development^2^, the time horizon, the inputs that are being used, and the expected impacts. Furthermore, it also describes the situation in which the intervention will (or will not) be implemented because of its (cost-) effectiveness. It is important that this phase counts with the participation of all the main stakeholders in the subject matter.

The next phase consists in defining the Markov model states. Those are defined based on the result of the main variable (outcome), in this case pollution (P), which could be measured with sensors. A hypothetical definition of the states could be:

- State A: extremely high pollution level (*P* = 40)^3^
- State B: slightly high pollution level (*P* = 30)
- State C: medium pollution level (*P* = 20)
- State D: low pollution level (*P* = 10)

The next step is to define the initial distribution of the population across the different states. In this case, the units are the neighborhoods where it is possible to measure the pollution level. We imagine that 40% are based on state A, 50% on state B, 9% on state C, and only 1% on state D.

Once the initial distribution has been completed, it is necessary to define the transition probabilities between states, for the *non-intervention* and *intervention* situations. This is the probability that after a certain period (e.g., 1 year), neighborhoods move from one pollution level to another. In terms of *ex post* evaluations, transition probabilities are calculated based on observed data. For *ex ante* evaluations, the probability of transition of the *intervention* can be based on studies of similar projects, whereas probability of transition *non-intervention* can be based on projections or actual trends. The following tables show a hypothetical situation; if there were 50 neighborhoods with a slightly high pollution level (state B), and it was estimated that in a year (with no actions taken) 30 of them would increase their pollution's level from slightly high to extremely high (state A), the probability of change from state B to state A in a *non-intervention* scenario would be 0.6. If it was estimated that five of those neighborhoods would move to a medium pollution level, probability of change from B to C would be 0.1. Finally, if it was expected that 15 neighborhoods would continue with the same pollution level, probability of staying at the same state B would be 0.3 (Table 1). It is necessary to keep in mind that the sum of all the probabilities located in the same row will always be one.

**Table 1:** Probabilities of transition between non-intervention states.

**Non-intervention**	**Transition a**			
**Initial state**	**State A**	**State B**	**State C**	**State D**
State A	0.9	0.1	0	0
State B	0.6	0.3	0.1	0
State C	0.2	0.4	0.4	0
State D	0	0	0.2	0.8

Implementing an intervention to promote public transportation, with the final goal of decreasing the pollution level, could influence the probabilities of transition. For example, of the 50 neighborhoods with slightly high pollution level, one could estimate that after a year of intervention, only 5 would increase their pollution level to extremely high, 15 would remain still, 20 would decrease their pollution level to medium, and 10 could achieve lower pollution level. In this case, probabilities of transition into A, C, and D states would be 0.1, 0.4, and 0.2, respectively, and 0.3 would be the probability of staying still on the same state B (Table 2). Therefore, intervention would increase the probabilities of transition into less polluted states. The same logic would be applied for neighborhoods that initially were in the other pollution states (A, C, and D).

**Table 2:** Probabilities of transition between intervention states.

**Intervention**	**Transition a**			
**Initial state**	**State A**	**State B**	**State C**	**State D**
State A	0.4	0.3	0.2	0.1
State B	0.1	0.3	0.4	0.2
State C	0.1	0.2	0.3	0.4
State D	0	0.1	0.3	0.6

Next, it is necessary to assign costs and effects to every state, both for *non-intervention* and *intervention* situations. In order to do this, implementation costs of the project (single costs) such as infrastructure (smart streetlights cost, electric charging stations costs, etc.), staff in charge of the installation, adaption and training costs, and bureaucratic costs must be quantified. In addition, necessary periodic costs must be calculated to ensure the proper function of the service (such as personnel in charge of providing the service, technical support staff, energy, Internet, management costs, etc.). On the other hand, in order to make the comparison possible, it is necessary to calculate the service costs applicable to that moment in time. Besides costs related directly with service provision, it is also useful to keep in mind any indirect costs. Sanitary costs of breathing illness due to pollution would be an example of this case.

Table 3 shows some input costs related to the intervention, whereas Table 4 shows a hypothetical sum of those elements. Besides that, Table 5 presents some possible indirect costs, which would depend on the level of pollution and which are therefore linked to each state. On the example, they are equal in both *intervention* and *non-intervention*, but they could differ in other situations. In that case, there would be the same number of sick people in state A in the case of *intervention* or *non-intervention*. However, the total number of sick people (and costs related to them) would be lower in the intervention scenario because there are more chances to transit to states less polluted, with less associated costs. Moreover, it is necessary to define correctly the unit that one uses to measure costs. In this case, all costs are homogenized per neighborhood (unit for this example) and per year^4^.

**Table 3:** Intervention costs.

	**Single costs**	**Periodic costs**
Non-intervention		Petrol cost
		Bus drivers' salary
Intervention	Sensor cost	Petrol cost
	Sensor cost installation	Bus drivers cost
	Training cost for drivers to	Personnel cost who monitor it
	adapt into new system	

*Inputs*.

**Table 4:** Intervention costs.

	**Single costs (per neighborhood)**	**Periodic costs (per neighborhood and year)**
Non-intervention	0	30 MU
Intervention	500 MU	20 MU

*Values*.

**Table 5:** Indirect costs (per neighborhood and year).

	**State A**	**State B**	**State C**	**State D**
Non-intervention	30 MU	25 MU	20 MU	15 MU
Intervention	30 MU	25 MU	20 MU	15 MU

*Values*.

Effects include direct results (related to the program) and indirect results (caused by attitudes and behavioral changes of the affected actors, effects above other sectorial areas of the city, etc.) They can be measured by monetary value, number of avoidable deaths, tons per person, etc. For the inclusion of more than one effect, the measures must be transformed into comparable units, for instance, applying percentages to define priority (environmental effects, effects that benefit the most vulnerable sectors, etc.). Regarding the example, the effects reflect how useful an intervention is to society, depending on the pollution level. It was assumed that less pollution was more useful, as it increases the quality of life and that it was equal to (100–C)/100. Consequently, costs and effects associated with each state would be the ones displayed in Table 6.

**Table 6:** Intervention and non-intervention costs and effects per state.

	**Intervention**		**Non-intervention**	
	**Costs**	**Effects**	**Costs**	**Effects**
State A	500 MU + 50 MU/year	0.6	60 MU/year	0.6
State B	500 MU + 45 MU/year	0.7	55 MU/year	0.7
State C	500 MU + 40 MU/year	0.8	50 MU/year	0.8
State D	500 MU + 35 MU/year	0.9	45 MU/year	0.9

## Results

**Figure 4:**
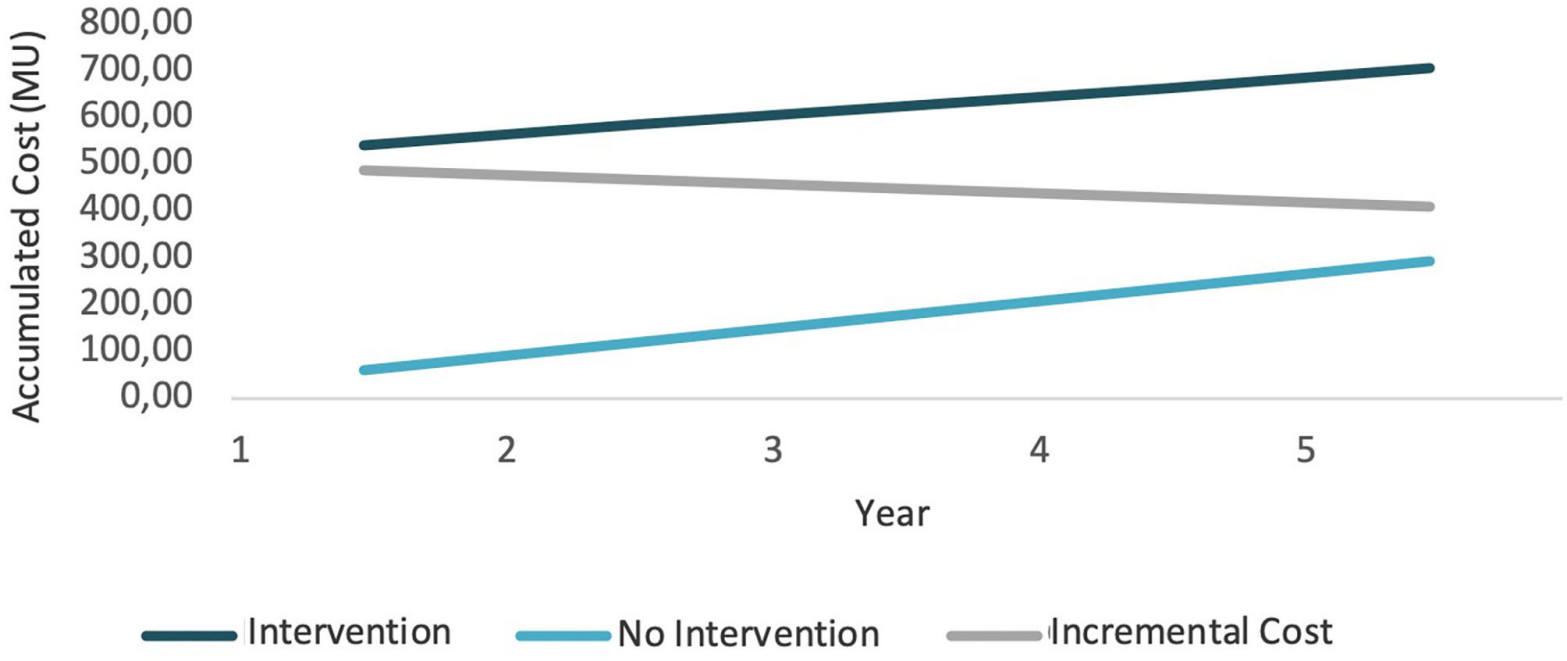
Cost evolution (1).

**Figure 5:**
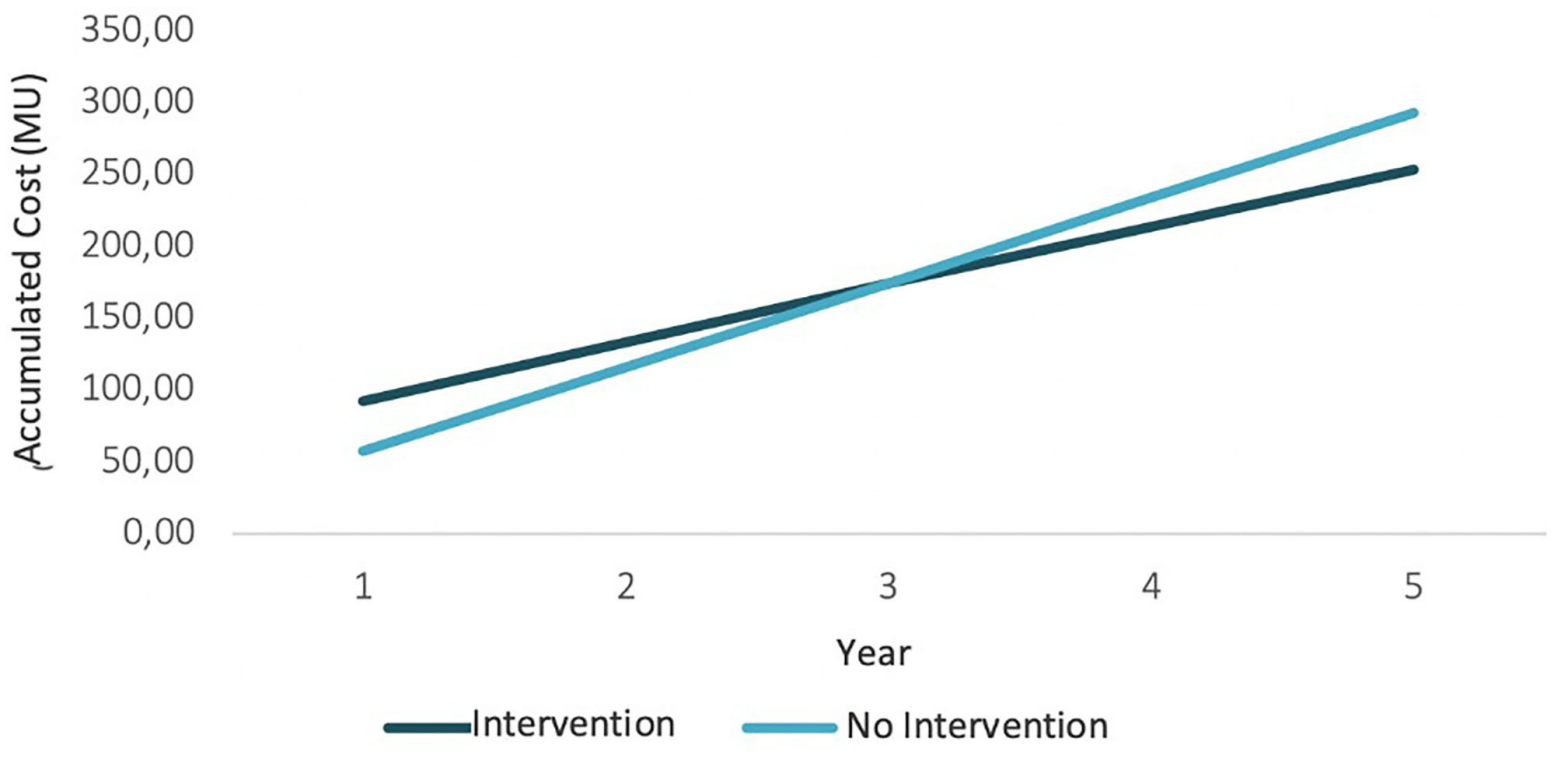
Cost evolution (2).

The results of the current case study showed that the intervention is more expensive than the non-intervention situation because of the initial investment. Nevertheless, if we look at the upcoming years, the difference between the non-intervention would be reduced because periodic costs would be smaller. At the same time, if the intervention succeeded in moving to states with less pollution, costs would also decrease, as states with less pollution have lower associated costs, especially if governments decide to tax greenhouse gas emissions. Figure 4 presents the cost evolution in both situations (intervention and non-intervention) for a 5-year period, where the intervention is more expensive than the non-intervention, but the difference between them is reduced. This can be seen on the incremental cost line. These results would change if intervention costs were lower. Figure 5 shows what would happen if initial investment costs were 50 MU instead of 500 MU. In that case, the intervention would initially be more expensive, but it would bring monetary savings afterward.

**Figure 6:**
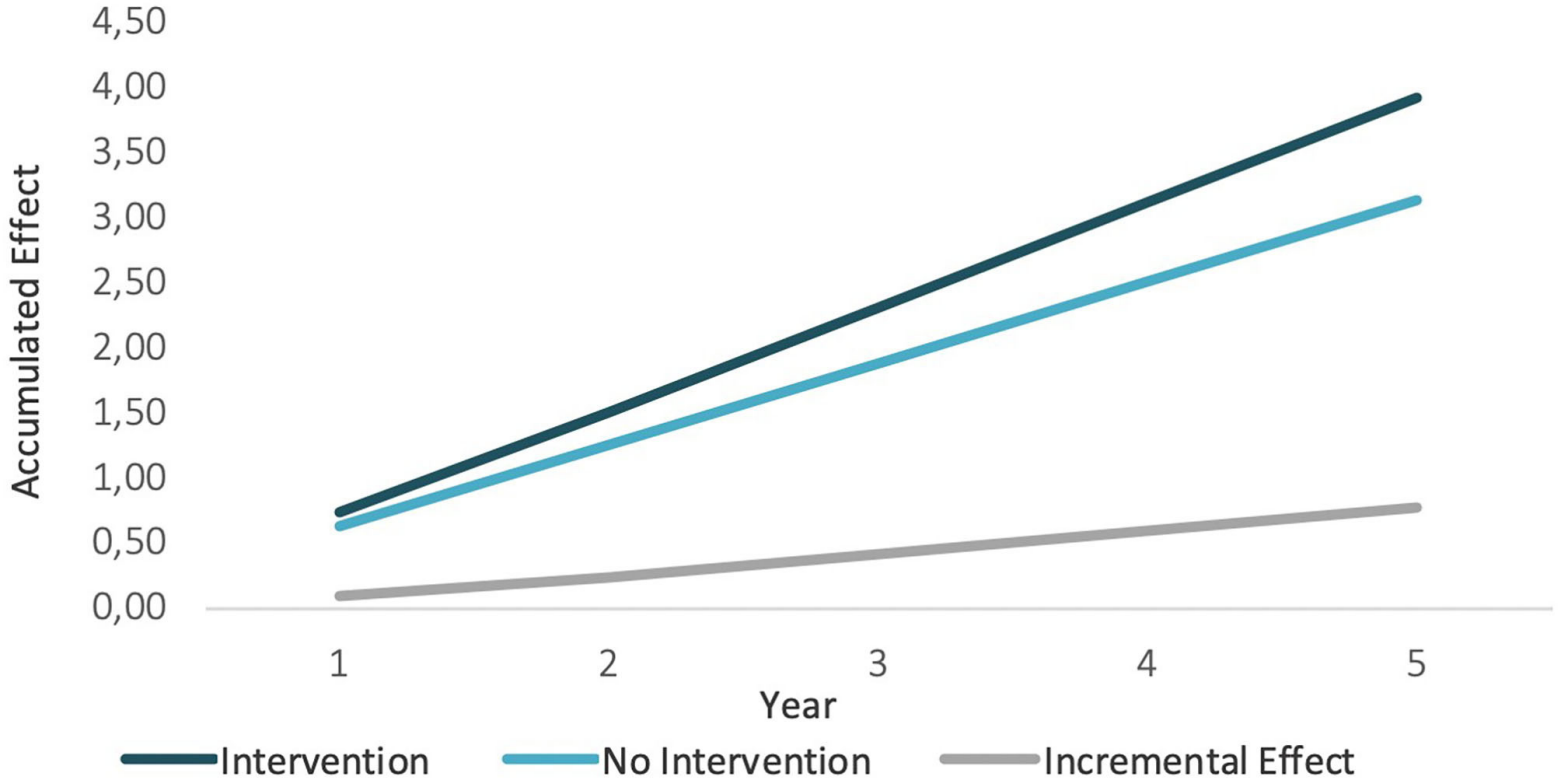
Incremental effects.

The other key results of the model are the effects derived from intervention, in this case the “usefulness” linked to the pollution level. Figure 6 shows how accumulative *intervention* effects increase faster than *non-intervention* effects (because there are more neighborhoods transiting to lower pollution levels), and consequently, the incremental effect is positive, and it increases.

**Figure 7:**
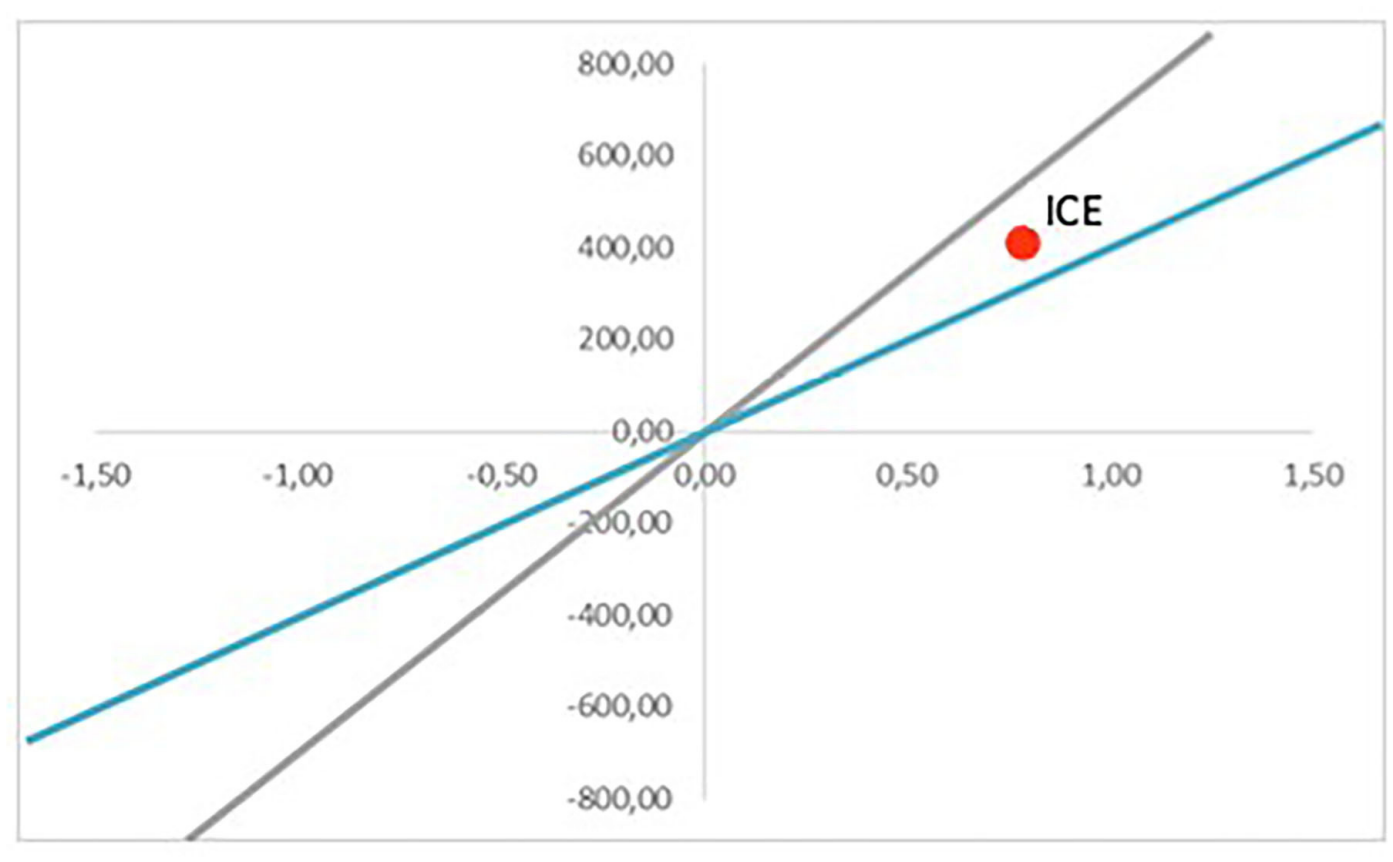
Cost-effectiveness plan (Scenario 1).

The final result of the evaluation is given by the ICE, which informs the decision making. Whether the program is implemented or not will depend on where the point is located. Figure 7 gives an example of a scenario where the necessary initial intervention investment is 500 MU. The horizontal axis represents the incremental effect, and the vertical axis represents the incremental cost. The ICE is located on the top right quadrant. Therefore, the WTP for reduction of the level of pollution will be the key to decide if the intervention will be implemented or not. If the WTP is 700 MU per usefulness unit (gray line), the intervention would be acceptable because the ICE would be below the threshold. However, if the WTP is 400 MU (blue line), the intervention should not be implemented. Therefore, in this scenario, the decision would depend on the WTP of the administration in charge of developing the project, which may depend on the available budget, the priority attributed to the impacts pursued by the intervention, etc. WTP also depends on the citizens or private organizations who will pay for the intervention in a direct or indirect way (through taxes). It should be noted that this WTP is not static, and the participation of the main stakeholders may be needed to define the limits in which an intervention would be considered acceptable.

**Figure 8:**
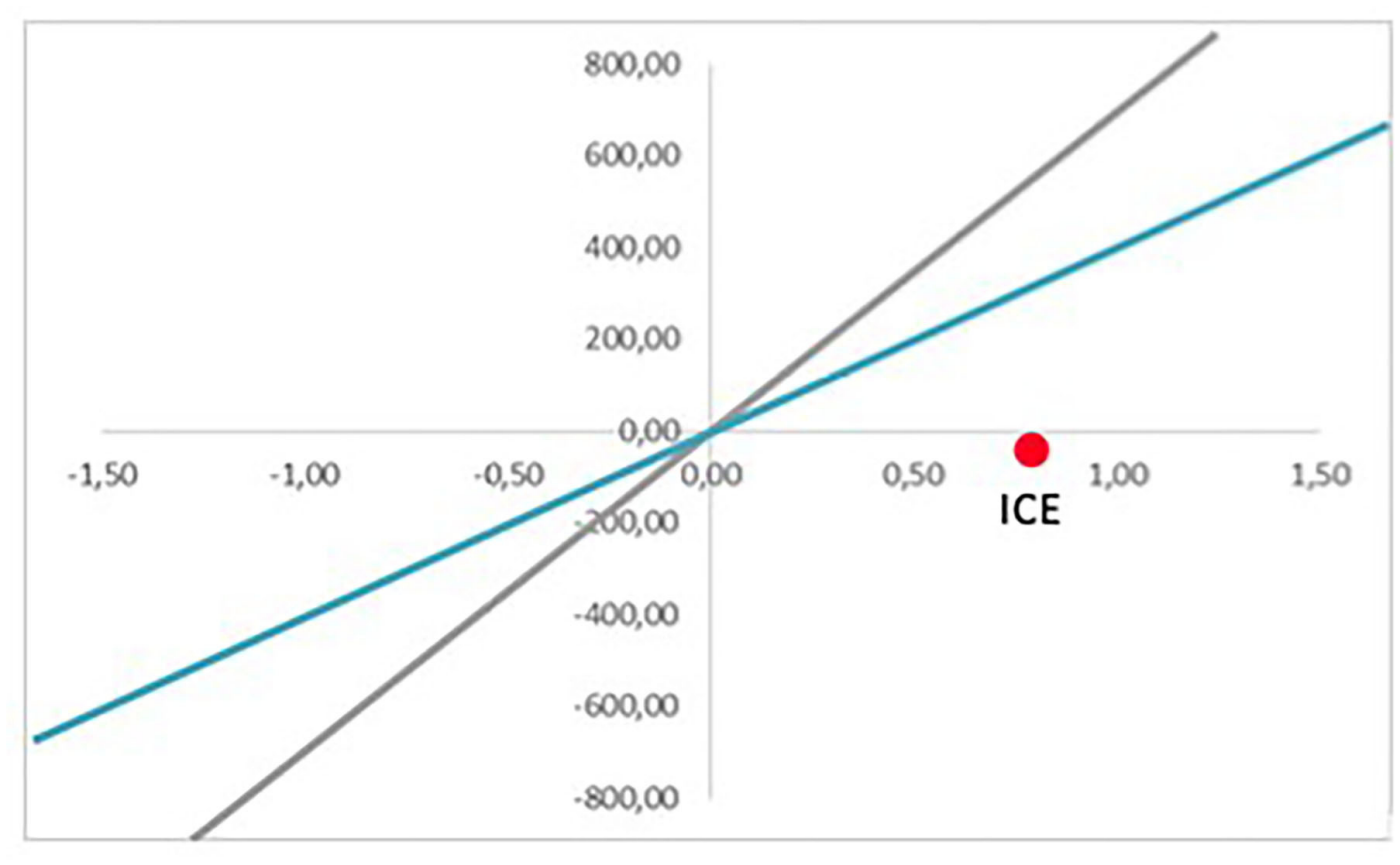
Cost-effectiveness plan (Scenario 2).

**Figure 9:**
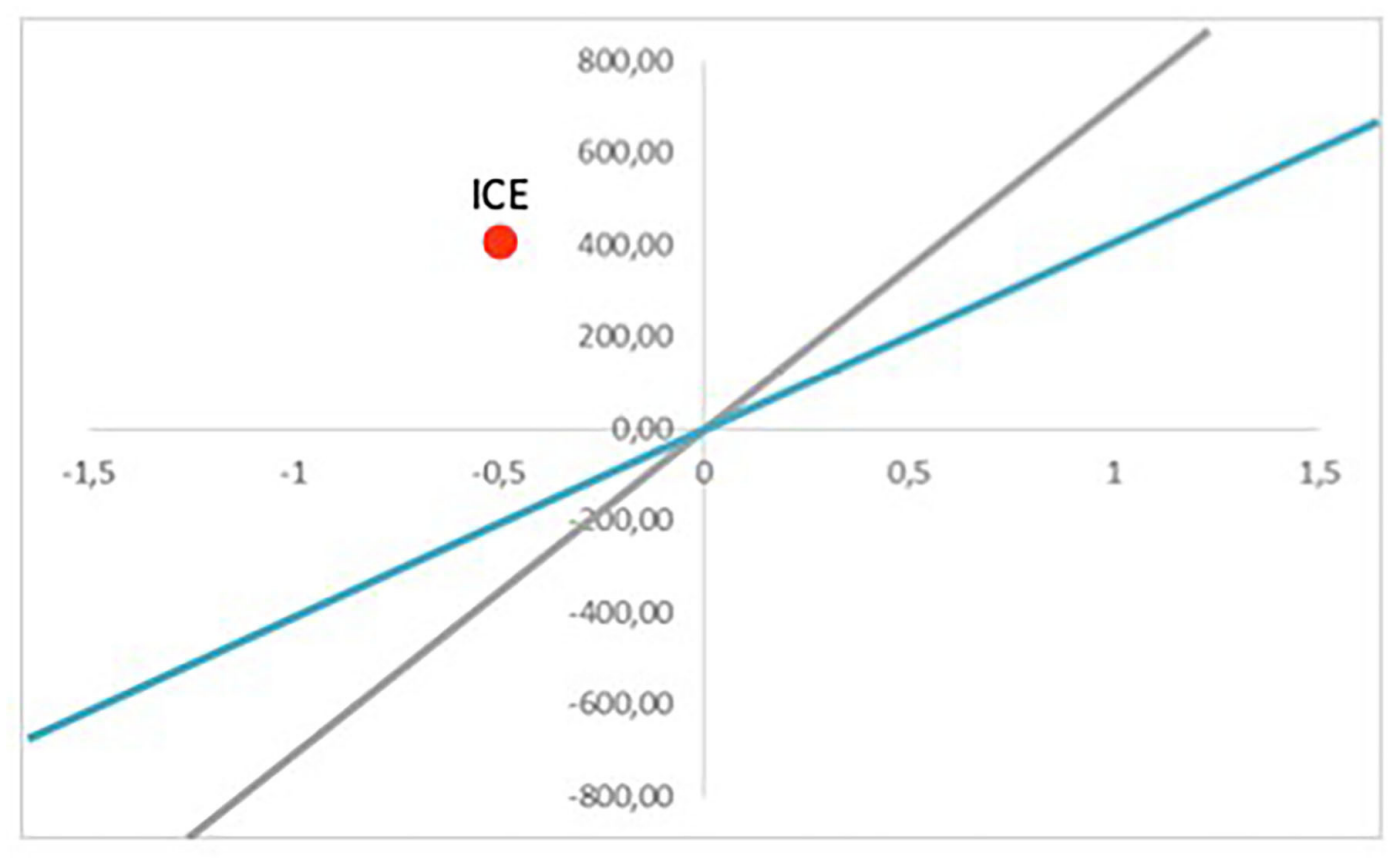
Cost-effectiveness plan (Scenario 3).

The simulation for Scenario 1 has been conducted under concrete parameters. However, these are just estimations, and they may deviate from reality. That is why the model can be run multiple times, demonstrating how results vary when certain parameters are updated. As seen previously, one change in the investment costs may modify the result of the accumulative costs. For example, for Scenario 2, the ICE would be in the bottom right quadrant, meaning the intervention is dominant. That means that it would be cheaper and more efficient than the current option, and therefore, it would be positive to implement it, regardless of WTP (Figure 8). On the other hand, Figure 9 brings a more pessimistic scenario in which the intervention would not be more effective than the current situation, and therefore, the final decision should be to not implement it. The different results would form the set of possible scenarios that could be reached by implementing a certain intervention, which would provide key information to the authorities in charge of making decisions.

## Discussion

Because of the improvement of healthcare delivery that makes healthy aging more possible than ever before, in addition to the growing world's population that has been moving toward urban areas over the past years, it has created the necessity to develop smart cities that are aware of the special needs of all their citizens (14, 15). Digital and smart technologies need to deliver affordable and easy-to-use solutions to a growing and aging population, thereby improving health and well-being of citizens (16). These developments, which are expected to keep happening within the next 40 years, bring great challenges and disadvantages associated with urban agglomerations. These challenges include several problems to be tackled from a policy point of view, such as resource scarcity, air pollution, decrease of citizen health, and traffic problems, among others (17). Thus, urban areas have increased their sizes, which has been made possible by a simultaneous upward shift in the urban technological frontier in order to allow cities to accommodate more inhabitants (18). The WHO Healthy Cities Network and associated national networks already have established hundreds of member cities globally that aim to benefit from IoT to improve the health and well-being of citizens, but assessing their full potential and impact is still scarce.

History shows that problems associated with urban agglomerations have been solved by different means related to creativity, human capital, and cooperation among stakeholders, which according to Kylili (18) can be called “smart” solutions. In this context, the European Union, as well as other international organizations, has put effort on devising a strategy to achieve urban growth in a smart way for its metropolitan areas, fostering development through information and communications technology–driven interventions, and improving citizens' health and well-being (14, 18). As a consequence, there has been an exponential growth of projects and other initiatives to develop and establish smart cities, creating a clear need for a reliable and valid methodology to evaluate these initiatives. The current innovative scenario demands a holistic methodological approach for assessing smart city projects that aim to improve health and well-being of citizens, which incorporates a combination of technologies and solutions that take into account progressive economic feasibility considerations (19). It is of utmost relevance for policy makers to adopt these methodologies, as they ensure the effectiveness of the interventions aiming at transitioning from the European cities as we know them into smart ones.

However, searches carried out have not identified any evaluation framework that is currently addressing this need. As we have shown in the present report, the MAFEIP tool is highly promising to support evidence-based decision making in the development and uptake of smart city interventions and technologies. MAFEIP goes beyond simple measurement with indicators such as those that we can find on many smart cities studies, to inform on the effects produced directly by an intervention and the expected impacts (*ex ante* evaluation). On their work, Caird (20) suggests that the selection of measurement indicators for evaluation purposes at project, program, and city levels and different geographical scales should be appropriate to smart city developments, with potential correspondences mapped between each level. The MAFEIP tool compares the costs and the efforts invested with the results to examine the profitability and the feasibility, giving smart cities interventions and technologies a chance to improve their efficiency and effectivity, scale up, and be able to improve decision making in this field.

## Conclusion

Evaluation plays a fundamental role in the development of smart city projects, as it is what allows decision-makers to identify programs that obtain the expected results. Additionally, the ex ante evaluation analyzes the expected results of one or more interventions before their application, in order to decide whether their implementation is recommended. For each intervention, MAFEIP analyzes how the costs are related to its effects, taking into account the WTP. The results can be positive or negative, of greater or lesser degree, and with a possible differential effect depending on the actors. MAFEIP allows the choice between alternatives that are normally exclusive due to budgetary restrictions and cannot be applied simultaneously. Thus, it is essential to make use of the evaluation before, during, and at the end of each public program or intervention with a public impact, like all those that fall within the framework of smart cities.

## Data Availability Statement

The raw data supporting the conclusions of this article will be made available by the authors, without undue reservation.

## Author Contributions

NF, FF, and FL-V have conceptualized the study design, designed the methodology, conducted the analyses, and wrote the manuscript. All authors have approved the final version of the manuscript and have approved the content.

## Conflict of Interest

The authors declare that the research was conducted in the absence of any commercial or financial relationships that could be construed as a potential conflict of interest.

## Footnotes

^1^It is also possible to use for more than two alternatives.

^2^These can be very diverse: stakeholder behavior, economic cycle, political context, capacity of the organization to implement the intervention, etc.

^3^“C” shows pollution levels.

^4^Depending on the evaluated intervention, the period could change.
